# Bell’s Nonlocality Can be Detected by the Violation of Einstein-Podolsky-Rosen Steering Inequality

**DOI:** 10.1038/srep39063

**Published:** 2016-12-14

**Authors:** Jing-Ling Chen, Changliang Ren, Changbo Chen, Xiang-Jun Ye, Arun Kumar Pati

**Affiliations:** 1 Theoretical Physics Division, Chern Institute of Mathematics, Nankai University, Tianjin 300071, People’s Republic of China; 2 Centre for Quantum Technologies, National University of Singapore, 3 Science Drive 2, Singapore 117543, Singapore; 3 Center for Nanofabrication and System Integration, Chongqing Institute of Green and Intelligent Technology, Chinese Academy of Sciences, Chongqing 400714, People’s Republic of China; 4 Chongqing Key Laboratory of Automated Reasoning and Cognition, Chongqing Institute of Green and Intelligent Technology, Chinese Academy of Sciences, Chongqing 400714, People’s Republic of China; 5 Key Laboratory of Quantum Information, University of Science and Technology of China, University of Science and Technology of China, Hefei 230026, People’s Republic of China; 6 Synergetic Innovation Center of Quantum Information and Quantum Physics, University of Science and Technology of China, Hefei 230026, People’s Republic of China; 7 Quantum Information and Computation Group, Harish-Chandra Research Institute, Chhatnag Road, Jhunsi, Allahabad 211019, India

## Abstract

Recently quantum nonlocality has been classified into three distinct types: quantum entanglement, Einstein-Podolsky-Rosen steering, and Bell’s nonlocality. Among which, Bell’s nonlocality is the strongest type. Bell’s nonlocality for quantum states is usually detected by violation of some Bell’s inequalities, such as Clause-Horne-Shimony-Holt inequality for two qubits. Steering is a manifestation of nonlocality intermediate between entanglement and Bell’s nonlocality. This peculiar feature has led to a curious quantum phenomenon, the one-way Einstein-Podolsky-Rosen steering. The one-way steering was an important open question presented in 2007, and positively answered in 2014 by Bowles *et al*., who presented a simple class of one-way steerable states in a two-qubit system with at least thirteen projective measurements. The inspiring result for the first time theoretically confirms quantum nonlocality can be fundamentally asymmetric. Here, we propose another curious quantum phenomenon: Bell nonlocal states can be constructed from some steerable states. This novel finding not only offers a distinctive way to study Bell’s nonlocality without Bell’s inequality but with steering inequality, but also may avoid locality loophole in Bell’s tests and make Bell’s nonlocality easier for demonstration. Furthermore, a nine-setting steering inequality has also been presented for developing more efficient one-way steering and detecting some Bell nonlocal states.

In 1935, the famous Einstein, Podolsky and Rosen (EPR) paper indicated that quantum mechanics is in conflict with the notion of locality and reality^1^. If local realism is correct, then quantum mechanics cannot be considered as a complete theory to describe physical reality. Immediately after the publication of the EPR paper, Schrödinger made a response by conjuring two important notions, namely, the quantum *entanglement* and the quantum *steering*. According to Schrödinger, quantum entanglement is “the characteristic trait of quantum mechanics” that distinguishes quantum theory from classical theory^2^. The notion of “steering” is closely related to the statement of “spooky action at a distance”, which Einstein was disturbed all the time. EPR steering reflects such a “spooky action” feature that manipulating one object seemingly affects another instantaneously, even it is far away.

Different to Schrödinger’s response, in 1964, Bell proposed an inequality for local hidden variable (LHV) models^3^. The violation of Bell’s inequality by quantum entangled states implies Bell’s nonlocality. This is well-known as Bell’s theorem, which has established what quantum theory can tell us about the fundamental features of *Natur*e, and been widely regarded as “the most profound discovery of science”^4^. Until now, the fundamental theorem has achieved ubiquitous applications in different quantum information tasks, such as quantum key distribution^5^, communication complexity^6^, and random number generation^7^.

Unlike quantum entanglement and Bell’s nonlocality, the research field of quantum steering has been sterile till 2007, when Wiseman, Jones, and Doherty^8^ reformulated the idea and placed it firmly on a rigorous ground. Since then EPR steering has gained a very rapid development in both theories^9^,^10^,^11^,^12^,^13^,^14^,^15^,^16^ and experiments^17^,^18^,^19^,^20^,^21^,^22^,^23^,^24^,^25^,^26^. Most research topics as well as research approaches in the field of Bell’s nonlocality have been transplanted similarly to the field of EPR steering. For instance, steering inequalities have been proposed to reveal the EPR steerability of quantum states, very similar to the violation of Bell’s inequalities reveals Bell’s nonlocality.

According to ref. 8, entanglement, EPR steering and Bell’s nonlocality are called by a joint name as “ quantum nonlocality”, which has an interesting hierarchical structure: quantum entanglement is a superset of steering, and Bell’s nonlocality is a subset of steering. However, among the three types of quantum nonlocality, only steering can possess a curious feature of “one-way quantumness”. Suppose Alice and Bob share a pair of two-qubit state, it is not hard to imagine that if Alice entangles with Bob, then Bob must also entangle with Alice. Such a symmetric feature holds for both entanglement and Bell nonlocality. However, the situation is dramatically changed when one turns to a novel kind of quantum nonlocality in the middle of entanglement and Bell nonlocality, the EPR steering. It may happen that for some asymmetric bipartite quantum states, Alice can steer Bob but Bob can never steer Alice. This distinguished feature would be useful for some one-way quantum information tasks, such as quantum cryptography. The “one-way EPR steering” or “asymmetric EPR steering” is an important “open question” first proposed by Wiseman *et al*. in ref. 8. Very recently, the question has been answered by Bowles *et al*.^15^, who presented a simple class of one-way steerable states in a two-qubit system with at least 13 projective measurements (a linear 14-setting steering inequality was given explicitly in the work). The inspiring result for the first time theoretically confirms quantum nonlocality can be fundamentally asymmetric. Later on, Bowles *et al*. investigated the one-way steering problem by presenting a sufficient criterion (being a nonlinear criterion) for guaranteeing that a two-qubit state is unsteerable^27^.

In this work, we focus on another curious quantum phenomenon raised by steering: Bell nonlocal states can be constructed from some EPR steerable states. Explicitly we present a theorem, showing that for any two-qubit state *τ*, if its corresponding state *ρ* is EPR steerable, then the state *τ* must be Bell nonlocal. Bell’s nonlocality of the quantum state *τ* can be detected indirectly by the violation of steering inequality for the quantum state *ρ*. The novel result not only pinpoints a deep connection between EPR steering and Bell’s nonlocality, but also sheds a new light to avoid locality loophole in Bell’s tests and make Bell’s nonlocality easier for demonstration. In addition, we also present a 9-setting linear steering inequality for developing more efficient one-way steering and detecting some Bell nonlocal states. We find that the new steering inequality can actually improve the result of ref. 15 by detecting the one-way steering with fewer measurement settings but with larger quantum violations, which would be helpful for the experimenters.

## Results

### Bell’s Nonlocal states can be constructed from EPR steerable states

**Figure 1:**
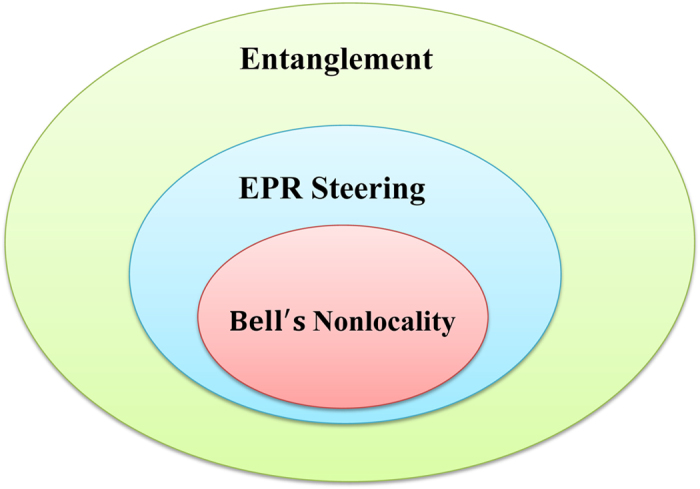
Hierarchical structure of quantum nonlocality. Bell’s nonlocality is the strongest type of quantum nonlocality. If a state possesses EPR steerability or Bell’s nonlocality, then the state must be entangled. EPR steering is a form of nonlocality intermediate between entanglement and Bell nonlocality.

**Figure 2:**
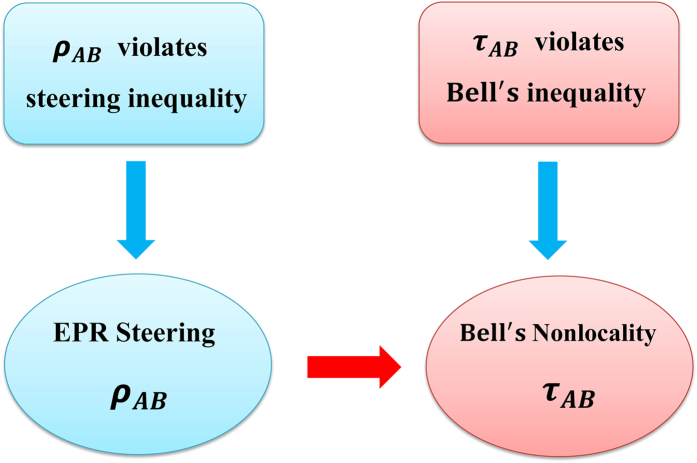
Illustration of detecting Bell’s nonlocality through EPR steering. If a state *ρ*_*AB*_ violates a steering inequality, then it implies that *ρ*_*AB*_ possesses the EPR steerability. Traditionally, Bell’s nonlocality of the two-qubit state *τ*_*AB*_ is revealed by violations of Bell’s inequality. Based on Theorem 1, Bell’s nonlocality of the state *τ*_*AB*_ can be detected through EPR steerability of the state *ρ*_*AB*_, and the relation between *ρ*_*AB*_ and *τ*_*AB*_ is given in Eq. (1).

It is well-known that quantum nonlocality possesses an interesting hierarchical structure (see Fig. 1). EPR steering is a weaker nonlocality in comparison to Bell’s nonlocality. Here we would like to pinpoint a curious quantum phenomenon directly connecting these two different types of nonlocality. We find that Bell’s nonlocal states can be constructed from some EPR steerable states, which indicates that Bell’s nonlocality can be detected indirectly through EPR steering (see Fig. 2), and offers a distinctive way to study Bell’s nonlocality. The result can be expressed as the following theorem.

**Theorem 1**: For any two-qubit state *τ*_*AB*_ shared by Alice and Bob, define another state


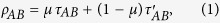


with 
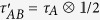
, 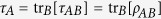 being the reduced density matrix at Alice’s side, and 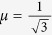. If *ρ*_*AB*_ is EPR steerable, then *τ*_*AB*_ is Bell nonlocal.

**Proof.** The implication of the theorem is that, the EPR steerability of the state *ρ*_*AB*_ determines Bell’s nonlocality of the state *τ*_*AB*_. Namely, the nonexistence of local hidden state (LHS) model for *ρ*_*AB*_ implies the nonexistence of LHV model for *τ*_*AB*_. We shall prove the theorem by proving its converse negative proposition: if the state *τ*_*AB*_ has a LHV model description, then the state *ρ*_*AB*_ has a LHS model description.

Suppose *τ*_*AB*_ has a LHV model description, then by definition for any projective measurements *A* for Alice and *B* for Bob, one always has the following relation


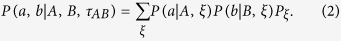


Here 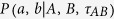 is the joint probability, quantum mechanically it is computed as 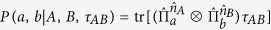, 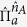 is the projective measurement along the 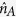-direction with measurement outcome *a* for Alice, 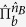 is the projective measurement along the 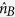-direction with measurement outcome *b* for Bob (with *a, b* = 0, 1), 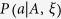, 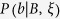 and *P*_*ξ*_ denote some (positive, normalized) probability distributions.

Let the measurement settings at Bob’s side be picked out as *x, y, z*. In this situation, Bob’s projectors are 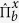, 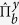, 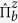, respectively. Since the state *τ*_*AB*_ has a LHV model description, based on Eq. (2) we explicitly have (with 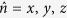)


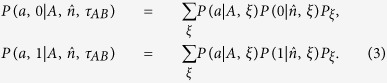


We now turn to study the EPR steerability of *ρ*_*AB*_. After Alice performs the projective measurement on her qubit, the state *ρ*_*AB*_ collapses to Bob’s conditional states (unnormalized) as


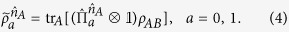


To prove that there exists a LHS model for *ρ*_*AB*_ is equivalent to proving that, for any measurement 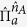 and outcome *a*, one can always find a hidden state ensemble 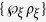 and the conditional probabilities 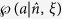, such that the relation


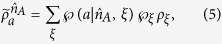


is always satisfied. Here *ξ*’s are the local hidden variables, *ρ*_*ξ*_’s are the hidden states, 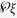 and 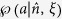 are probabilities satisfying 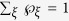 and 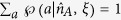. If there exist some specific measurement settings of Alice, such that Eq. (5) cannot be satisfied, then one must conclude that the state *ρ*_*AB*_ is steerable (in the sense of Alice steers Bob’s particle).

Suppose there is a LHS model description for *ρ*_*AB*_, then it implies that, for Eq. (5) one can always find the solutions of 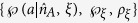 if Eq. (3) is valid. The solutions are given as follows:


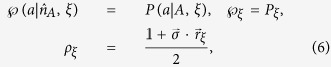


where 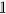 is the 2 × 2 identity matrix, 
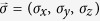
 is the vector of the Pauli matrices, and the hidden state *ρ*_*ξ*_ has been parameterized in the Bloch-vector form, with


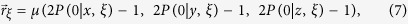


which is the Bloch vector for density matrix of a qubit. It can be checked that 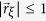, and this ensures *ρ*_*ξ*_ being a density matrix.

By substituting Eq. (6) into Eq. (5), we obtain


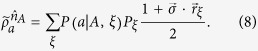


To prove the theorem is to verify the relation (8) is always satisfied if Eq. (3) is valid. The verification can be found in **Methods**.

*Remark 1*.— In Eq. (7), by requiring the condition 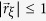 be valid for any probabilities *P*(0|*x, ξ*), *P*(0|*y, ξ*), *P*(0|*z, ξ*) ∈ [0, 1], in general one can have 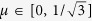. Generally, Theorem 1 is valid for any 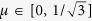. In the theorem we have chosen the parameter *μ* as its maximal value 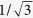, because the state *τ*_*AB*_ is convexed with a separable state 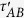, the larger value of *μ*, the easier to detect the EPR steerability.

In the following, we provide two examples for the theorem, showing that Bell’s nonlocality of quantum states can be detected indirectly by the violations of some steering inequalities.

*Example 1*.— For example, let us detect Bell’s nonlocality of the maximally entangled state (with *τ*_*AB*_ = |Ψ〉 〈Ψ|)


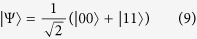


without Bell’s inequality. Based on the theorem, it is equivalent to detect the EPR steerability of the following two-qubit state


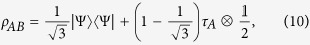


with 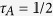. The state (10) is nothing but the Werner state^28^ with the visibility equals to 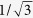, its steerability can be tested by using the steering inequality proposed in ref. 17 as


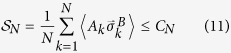


with *N* = 6. Here 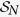 is the steering parameter for *N* measurement settings, and *C*_*N*_ is the classical bound, with 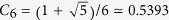. The maximal quantum violation of the steering inequality is 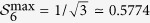, which beats the classical bound.

*Remark 2*.— In a two-qubit system, Bell’s nonlocality is usually detected by quantum violation of the Clause-Horne-Shimony-Holt inequality^29^. Bell’s nonlocality is the strongest type of nonlocality, due to this reason Bell-test experiments have encountered both the locality loophole and the detection loophole for a very long time^30^. As a weaker nonlocality, EPR steering naturally escapes from the locality loophole and is correspondingly easier to be demonstrated without the detection loophole^19^,^20^, as stated in ref. 17: “*because the degree of correlation required for EPR steering is smaller than that for violation of a Bell inequality, it should be correspondingly easier to demonstrate steering of qubits without making the fair*-*sampling assumption* [i.e., closing the detection loophole]”. Indeed, the steerability of the Werner state has been experimentally detected in ref. 17 by the steering inequality (11). Our result shows that the EPR steerability of the state *ρ*_*AB*_ determines Bell’s nonlocality of the state *τ*_*AB*_, thus may shed a new light to realize a loophole-free Bell-test experiment through the violation of steering inequality.

*Example 2*.— The theorem naturally provides a steering-based criterion for Bell’s nonlocality, which is expressed as follows: given an EPR steerable two-qubit state *ρ*_*AB*_, if the matrix


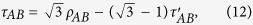


is a two-qubit density matrix, then *τ*_*AB*_ is Bell nonlocal.

Let us consider a two-qubit state *ρ*_*AB*_ in the following form


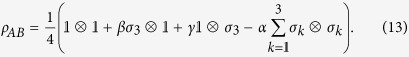


By substituting the state *ρ*_*AB*_ as in Eq. (13) into Eq. (12), then one obtains


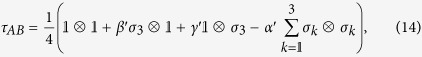


with


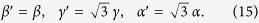


It is worth to mention that the steering inequality (11) is applicable to show Bell’s nonlocality of *τ*_*AB*_ for some parameters *α*′, *β*′, *γ*′. Here we would like to show that the similar task can be done by other new steering inequalities. In the following, we present a 9-setting linear steering inequality as


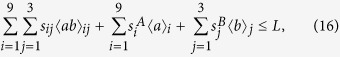


here for convenient we have used the same notations as in ref. 15 (where (*σ*_1_, *σ*_2_, *σ*_3_) is equivalent to (*σ*_*x*_, *σ*_*y*_, *σ*_*z*_)). The inequality are characterized by matrices {**S**, **S**^**A**^, **S**^**B**^} with real coefficients *s*_*ij*_, 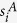, and 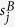, and the local bound is *L* = 1 (see Supplementary Materials). The steering inequality (16) may have other particular application for improving the result ref. 15 by developing more efficient one-way steering, which we shall address in the coming section. But now we use it to detect Bell’s nonlocality.

For example, let *α*′ = 0.96, *β*′ = −1/5, *γ*′ = 1/6, ones finds that *τ*_*AB*_ is a two-qubit state, and the steering inequality (16) is violated by the state *ρ*_*AB*_ (with the violation value 1.0064), hence the Bell’s nonlocality of state *τ*_*AB*_ can be revealed in this way indirectly by the steerability of the state *ρ*_*AB*_.

### More efficient one-way EPR steering

Under local unitary transformation (LUT), any two-qubit state can be written in the following form ref. 31


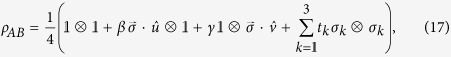


with *β, γ, t*_*k*_ being the real coefficients, and 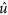, 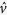 the unit vectors. Obviously, under LUT, the state *ρ*_*AB*_ is said to be symmetric if and only if *β* = *γ* and 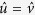. Let one consider a simple situation with *t*_1_ = *t*_2_ = *t*_3_ = −*α*, and 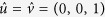, then he obtains the two-qubit state *ρ*_*AB*_ as in Eq. (13). In such a case, if *ρ*_*AB*_ is a one-way steerable state, then one must have *β* ≠ *γ*.

In ref. 15, the authors have chosen 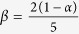, 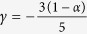 and used the SDP program to numerically prove that the state *ρ*_*AB*_ is a one-way steerable state (with at least 13 projective measurements): for *α* ≤ 1/2, the state *ρ*_*AB*_ is unsteerable from Bob to Alice, while for 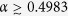 the state is steerable from Alice to Bob when Alice performs 14 projective measurements. An explicit 14-setting steering inequality has been also proposed to conform the one-way steerability, although for *α* = 1/2, the quantum violation is tiny (only 1.0004). The inspiring result for the first time confirms that the nonlocality can be fundamentally asymmetric. However, the tiny inequality violation as well as the 14 measurement settings give rise to the difficulty in experimental detection. To advance the study of unidirectional quantum steering, here we present a more efficient class of one-way steerable states by choosing


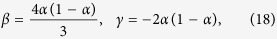


with *α* ∈ [0, 1]. The state *ρ*_*AB*_(*α*) is entangled for *α* > 0.3279. With the help of the SDP program, we found that in the range 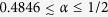, the state *ρ*(*α*) is one-way steerable within 10-setting measurements, thus this is more efficient than the previous result in ref. 15 (For the detail derivation of more efficient one-way EPR steering see Supplementary Materials). Furthermore, we can extract an explicit 9-setting steering inequalities (16) based on the SDP program. It can be verified directly that, for the state *ρ*_*AB*_(1/2), the quantum violation of 9-setting inequality (16) is 
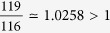
, hence demonstrating steering from Alice to Bob. Compared to the previous result^15^, the amount of violation is much larger but achieved with fewer measurements. To our knowledge, we do not know whether the quantum violation by inequality (16) could be observed with current quantum technology. However, we believe that this result would be interesting and helpful for both theoretical and experimental physicists.

## Discussion

In this work, we have presented a theorem showing that Bell nonlocal states can be constructed from some EPR steerable states. This result not only offers a novel and distinctive way to study Bell’s nonlocality with the violation of steering inequality, but also may avoid locality loophole in Bell’s tests and make Bell’s nonlocality easier for demonstration. An interesting and inverse problem is whether one can construct some steerable states *τ*_*AB*_ from some Bell nonlocal state *ρ*_*AB*_, because Bell’s nonlocality has been researched more deeply in theoretical aspect, so that people can conveniently study steering via known criteria of Bell’s nonlocality. Furthermore, an explicit 9-setting linear steering inequality has also been presented for detecting some Bell nonlocal states and developing more efficient one-way steering. This result allows one to observe one-way EPR steering with fewer measurement setting but with larger quantum violations. We hope experimental progress in this direction could be made in the near future.

## Methods

### Verification of **equation (8)**

Let us calculate the left-hand side of Eq. (8). One has


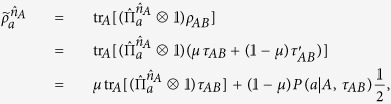


where 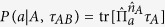 is the marginal probability of Alice when she measures *A* and gets the outcome *a*. For convenient, let us denote the 2 × 2 matrix 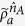 as


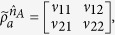


and calculate its each element. We get


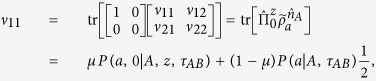


and similarly,


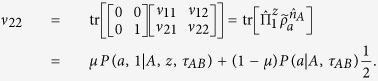


Note that 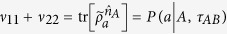, we then have


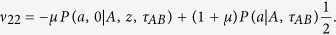


Because


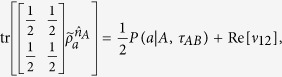


with Re[*ν*_12_] is the real part of *ν*_12_, thus,


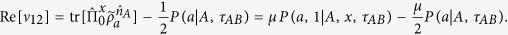


Similarly, because


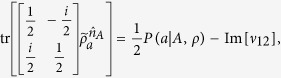


with Im[*ν*_12_] is the imaginary part of *ν*_12_, thus,


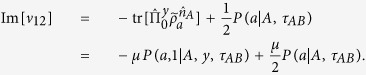


By combining the above equations, we finally have


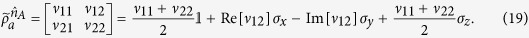


Let us calculate the right-hand side of Eq. (8). It gives


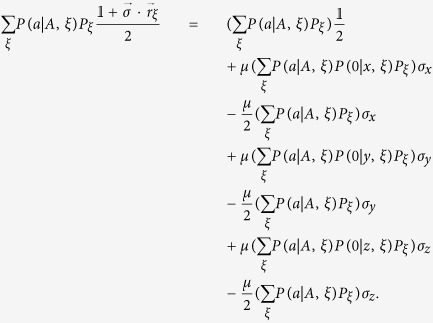


With the help of Eq. (3) and using 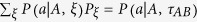, we finally have


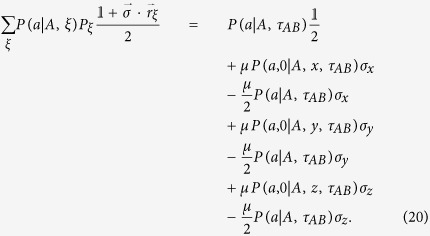


By comparing Eqs (19) and (20), it is easy to see that Eq. (8) holds. Thus, if there is a LHV model description for *τ*_*AB*_, then there is a LHS model description for *ρ*_*AB*_. This completes the proof.

## Additional Information

**How to cite this article**: Chen, J.-L. *et al*. Bell’s Nonlocality Can be Detected by the Violation of Einstein-Podolsky-Rosen Steering Inequality. *Sci. Rep.* **6**, 39063; doi: 10.1038/srep39063 (2016).

**Publisher's note:** Springer Nature remains neutral with regard to jurisdictional claims in published maps and institutional affiliations.

## Supplementary Material

Supplementary Information
