# Supplementary material for: Bell’s Nonlocality Can be Detected by the Violation of Einstein-Podolsky-Rosen Steering Inequality
Source: Sci Rep. 2016 Dec 14;6:39063. doi: 10.1038/srep39063 (PMC5155282; doi:10.1038/srep39063)
Supplement: Supplementary Information [file srep39063-s1.pdf]

Supplementary Materials

for "Bell's Nonlocality Can be Detected by the Violation of Einstein-Podolsky-Rosen Steering Inequality"

Jing-Ling Chen,[1,2] Changliang Ren,[3] Changbo Chen,[4] Xiang-Jun Ye,[5,6] and Arun Kumar Pati[7]

[1]*Theoretical Physics Division, Chern Institute of Mathematics, Nankai University, Tianjin 300071, People's Republic of China*
[2]*Centre for Quantum Technologies, National University of Singapore, 3 Science Drive 2, Singapore 117543, Singapore*
[3]*Center for Nanofabrication and System Integration, Chongqing Institute of Green and Intelligent Technology, Chinese Academy of Sciences, Chongqing 400714, People's Republic of China*
[4]*Chongqing Key Laboratory of Automated Reasoning and Cognition, Chongqing Institute of Green and Intelligent Technology, Chinese Academy of Sciences, Chongqing 400714, People's Republic of China*
[5]*Key Laboratory of Quantum Information, University of Science and Technology of China, University of Science and Technology of China, Hefei 230026, People's Republic of China*
[6]*Synergetic Innovation Center of Quantum Information and Quantum Physics, University of Science and Technology of China, Hefei 230026, People's Republic of China*
[7]*Quantum Information and Computation Group, Harish-Chandra Research Institute, Chhatnag Road, Jhunsi, Allahabad 211019, India*
(Dated: November 10, 2016)

I. DETAIL DERIVATION OF MORE EFFICIENT ONE-WAY EPR STEERING

In Ref. [1], Bowles, Vertesi, Quintino, and Brunner (BVQB) have presented a class of one-parameter two-qubit state

$$\rho_{AB}(\alpha)=\alpha|\psi^-\rangle\langle\psi^-|+\frac{1-\alpha}{5}\left(2|0\rangle\langle 0|\otimes\frac{\mathbb{1}}{2}+3\frac{\mathbb{1}}{2}\otimes|1\rangle\langle 1|\right), \tag{1}$$

where

$$|\psi^-\rangle=\frac{1}{2}(|01\rangle-|10\rangle) \tag{2}$$

is the singlet state and the parameter

$$\alpha\in[0,1]. \tag{3}$$

It is easy to find that the state (1) is identical to the following form of the two-qubit density matrix

$$\rho_{AB}=\frac{1}{4}\left(\mathbb{1}\otimes\mathbb{1}+\beta\sigma_3\otimes\mathbb{1}+\gamma\mathbb{1}\otimes\sigma_3-\alpha\sum_{k=1}^{3}\sigma_k\otimes\sigma_k\right). \tag{4}$$

with the specific values

$$\beta=\frac{2(1-\alpha)}{5},\quad \gamma=-\frac{3(1-\alpha)}{5}. \tag{5}$$

However, BVQB did not mention where the state (1) came from and how to construct it. Here, we provide a detail derivation, and from the derivation one can naturally achieve some more efficient states for demonstrating one-way EPR steering.

The derivation is just based on the BVQB LHS model, in which Bob can never steer Alice with any measurement settings (see the section "*No steering from B to A*" in [1]). In the BVQB model, supposed that Alice chooses an arbitrary measurement direction $\vec{x}=(x_1,x_2,x_3)$, and $\vec{y}=(y_1,y_2,y_3)$ for Bob, then from the viewpoint of LHS, the local expectation values and the correlation are given by

$$\begin{aligned}\langle a\rangle_{\rm LHS}&=\frac{x_3}{3},\\ \langle b\rangle_{\rm LHS}&=-\frac{y_3}{2},\\ \langle ab\rangle_{\rm LHS}&=-\frac{\vec{x}\cdot\vec{y}}{2}.\end{aligned} \tag{6}$$

Under local unitary transformation (LUT), any two-qubit state can be written in the following form [2]

$$\rho_{AB} = \frac{1}{4}\left(\mathbb{1}\otimes\mathbb{1} + \beta\,\vec{\sigma}\cdot\hat{u}\otimes\mathbb{1} + \gamma\,\mathbb{1}\otimes\vec{\sigma}\cdot\hat{v} + \sum_{k=1}^{3} t_k\sigma_k\otimes\sigma_k\right), \tag{7}$$

with β, γ, t_k being the real coefficients, and $\hat{u}, \hat{v}$ the unit vectors. During demonstrating the steerable states, we may ask a reverse question: for the BVQB model, which quantum states can be described by it? Without loss of generality, we can analyze how to extract these unsteerable states that can be described by the BVQB model from an arbitrary two-qubit state (7). The necessary condition is that the quantum expectation values of arbitrary measurement directions should coincident with those of the BVQB model. Obviously, the joint and marginal expectation values derived from quantum mechanics should satisfy the following relations:

$$\begin{aligned}
\langle a\rangle_{\rm QM} &= {\rm tr}[\rho_{AB}(\vec{\sigma}\cdot\vec{x}\otimes\mathbb{1})] \sim x_3,\\
\langle b\rangle_{\rm QM} &= {\rm tr}[\rho_{AB}(\mathbb{1}\otimes\vec{\sigma}\cdot\vec{y})] \sim y_3,\\
\langle ab\rangle_{\rm QM} &= {\rm tr}[\rho_{AB}(\vec{\sigma}\cdot\vec{x}\otimes\vec{\sigma}\cdot\vec{y})] \sim \vec{x}\cdot\vec{y}.
\end{aligned} \tag{8}$$

This condition is satisfied if and only if $t_1 = t_2 = t_3 = -\alpha$ and $\hat{u} = \hat{v} = (0, 0, 1)$, hence from the state (7) we arrive at

$$\rho_{AB} = \frac{1}{4}\left(\mathbb{1}\otimes\mathbb{1} + \beta\sigma_3\otimes\mathbb{1} + \gamma\mathbb{1}\otimes\sigma_3 - \alpha\sum_{k=1}^{3}\sigma_k\otimes\sigma_k\right) \tag{9}$$

which is just (4).

For the state $\rho_{AB}(1/2)$ in (1) (It is sufficient to consider $\rho_{AB}(\alpha)$ with $\alpha = 1/2$. The extension of the case $\alpha \leq 1/2$ is straightforward [1]), one can have the joint and marginal quantum expectation values as

$$\begin{aligned}
\langle a\rangle_{\rm QM} &= {\rm tr}[\rho_{AB}(\vec{\sigma}\cdot\vec{x}\otimes\mathbb{1})] = \frac{x_3}{5},\\
\langle b\rangle_{\rm QM} &= {\rm tr}[\rho_{AB}(\mathbb{1}\otimes\vec{\sigma}\cdot\vec{y})] = -\frac{3y_3}{10},\\
\langle ab\rangle_{\rm QM} &= {\rm tr}[\rho_{AB}(\vec{\sigma}\cdot\vec{x}\otimes\vec{\sigma}\cdot\vec{y})] = -\frac{\vec{x}\cdot\vec{y}}{2}.
\end{aligned} \tag{10}$$

However, Eq. (6) cannot simulate directly the quantum values expressed in Eq. (10). To do this, BVQB introduced a parameter of flipping probability f with

$$f \in [0, 1/2], \tag{11}$$

then Eq. (6) becomes

$$\begin{aligned}
\langle a\rangle_{\rm LHS} &= \frac{1-2f}{3}x_3,\\
\langle b\rangle_{\rm LHS} &= -\frac{1-2f}{2}y_3,\\
\langle ab\rangle_{\rm LHS} &= -\frac{\vec{x}\cdot\vec{y}}{2}.
\end{aligned} \tag{12}$$

By choosing $f = 1/5$, Eq. (6) exactly simulates the quantum results in Eq. (10), thus proving $\rho_{AB}(1/2)$ is unsteerable from Bob to Alice.

Let us return to the state (9), quantum mechanically one can have

$$\begin{aligned}
\langle a\rangle_{\rm QM} &= {\rm tr}[\rho_{AB}(\vec{\sigma}\cdot\vec{x}\otimes\mathbb{1})] = \beta\,x_3,\\
\langle b\rangle_{\rm QM} &= {\rm tr}[\rho_{AB}(\mathbb{1}\otimes\vec{\sigma}\cdot\vec{y})] = \gamma\,y_3,\\
\langle ab\rangle_{\rm QM} &= {\rm tr}[\rho_{AB}(\vec{\sigma}\cdot\vec{x}\otimes\vec{\sigma}\cdot\vec{y})] = -\alpha\,\vec{x}\cdot\vec{y}.
\end{aligned} \tag{13}$$

By comparing the first two formulae in Eq. (12) and Eq. (13) one has

$$\beta = \frac{1-2f}{3}, \quad \gamma = -\frac{1-2f}{2}. \tag{14}$$

Submitting Eq. (14) into Eq. (9) one has a two-parameter quantum state as

$$\rho_{AB}(\alpha, f) = \frac{1}{4}\left(\mathbb{1} \otimes \mathbb{1} + \frac{(1-2f)}{3}\, \sigma_3 \otimes \mathbb{1} - \frac{(1-2f)}{2}\, \mathbb{1} \otimes \sigma_3 - \alpha \sum_{k=1}^{3} \sigma_k \otimes \sigma_k\right). \tag{15}$$

Remark 1.— If one chooses

$$f = 1/2, \tag{16}$$

then one has $\beta = \gamma = 0$. In this case, the state (15) is a symmetric state, and it is just the Werner state. It is well-known that the Werner state is unsteerable for the region $\alpha \leq 1/2$ [3].

Remark 2.— By comparing Eq. (5) and Eq. (14), one obtains

$$f = \frac{3}{5}\left(\alpha - \frac{1}{6}\right). \tag{17}$$

Namely, if one selects the parameter f as a linear function of α as $f(\alpha) = 3(\alpha - 1/6)/5$, then one recovers the BVQB state as in Eq. (1). If α runs from $1/6$ to 1, the parameter f will run from 0 to $1/2$, and $\alpha = 1/2$ corresponds to $f = 1/5$. For the state $\rho_{AB}(\alpha = 1/2, f = 1/5)$, Ref. [1] has proved that with at lease 13 projective measurements Alice can steer Bob's qubit state. An explicit 14-setting steering inequality has also been proposed in Ref. [1] to conform the one-way steerability, although for $\alpha = 1/2$, the quantum violation is tiny (only $2269/2268 \backsimeq 1.0004$). The inspiring result for the first time confirms that the nonlocality can be fundamentally asymmetric. However, the tiny inequality violation as well as the 14 measurement settings give rise to the difficulty in experimental detection. By the way, the BVQB LHS model is not valid for the region of $\alpha \in [0, \frac{1}{6}]$, however in this case $\rho_{AB}(\alpha)$ is a separable state that can easily have other description of LHS models.

Remark 3.— We now come to extract some more efficient one-way steerable states from Eq.(15). For convenient to compare with the result of Ref. [1], here we also present a one-parameter quantum state by choosing

$$f = 2\left(\alpha - \frac{1}{2}\right)^2. \tag{18}$$

Explicitly, the state is given by

$$\rho_{AB}(\alpha) = \frac{1}{4}\left(\mathbb{1} \otimes \mathbb{1} + \frac{4\alpha(1-\alpha)}{3}\, \sigma_3 \otimes \mathbb{1} - 2\alpha(1-\alpha)\, \mathbb{1} \otimes \sigma_3 - \alpha \sum_{k=1}^{3} \sigma_k \otimes \sigma_k\right). \tag{19}$$

For the selection of the parameter f in Eq. (18), there are three reasons: (i) similar to the state presented in Ref. [1], the matrix $\rho_{AB}(\alpha)$ in Eq. (19) is always a density matrix for the all region of $\alpha \in [0, 1]$; (ii) when α runs from 0 to 1, the parameter f always stays in the region of $[0, 1/2]$, such that if $\rho_{AB}(\alpha = 1/2)$ has a description of the BVQB LHS model, then it ensures that $\rho_{AB}(\alpha < 1/2)$ also has a description of the BVQB model; (iii) $\rho_{AB}(\alpha)$ is a more efficient one-way steerable state, as we shall show below.

First, based on the BVQB model, no steering from Bob to Alice for the state $\rho_{AB}(\alpha)$ when $\alpha \leq 1/2$. Second, we need to show Alice can steer Bob with the state $\rho_{AB}(\alpha \leq 1/2)$. We shall show that the state $\rho(\alpha)$ with $\alpha \gtrsim 0.4846$ is steerable from Alice to Bob. With the help of the SDP program, we have calculated the threshold values α^* for which the state $\rho(\alpha)$ is steerable from Alice to Bob for different m measurement directions $\vec{x}$ with $m = 2, 3, ..., 10$ (see Table I). Definitely, for $m = 8$ we obtain $\alpha^* \backsimeq 0.4982$, thus implying that the state $\rho(\alpha)$ with $\alpha^* \gtrsim 0.4982$ is steerable from Alice to Bob. Hence it shows a class of more efficient one-way steerable states than that of Ref. [1], in which the states with $\alpha^* \gtrsim 0.4983$ are steerable from Alice to Bob when Alice performs 14 projective measurements. And for $m = 10$, we obtain a larger value of $\alpha^* \backsimeq 0.4846$.

m	2	3	4	5	6	7	8	9	10
α^*	0.6302	0.5461	0.5244	0.5147	0.5071	0.5041	0.4982	0.4855	0.4846

TABLE I: Threshold values α^* for which the state $\rho(\alpha)$ is steerable from Alice to Bob, when Alice performs $m = 2, 3, ..., 10$ projective measurements on her qubit, respectively.

Third, we can extract analytic 8-setting and 9-setting linear steering inequalities based on SDP program. For example, the 8-setting steering inequality is given by

$$\sum_{i=1}^{8} \sum_{j=1}^{3} s_{ij} \langle ab \rangle_{ij} + \sum_{i=1}^{8} s_i^A \langle a \rangle_i + \sum_{j=1}^{3} s_j^B \langle b \rangle_j \leq L, \tag{20}$$

with the local bound $L = 1$, and

$$\mathbf{S} = \begin{pmatrix} -\frac{1}{75} & -\frac{6}{43} & -\frac{13}{86} \\ -\frac{16}{87} & \frac{9}{157} & \frac{13}{111} \\ -\frac{1}{314} & -\frac{15}{116} & -\frac{1}{35} \\ -\frac{18}{103} & \frac{8}{67} & -\frac{1}{46} \\ -\frac{2}{49} & \frac{15}{79} & -\frac{16}{109} \\ \frac{15}{119} & \frac{15}{133} & -\frac{14}{79} \\ \frac{5}{117} & -\frac{1}{104} & \frac{13}{60} \\ -\frac{22}{103} & -\frac{27}{242} & -\frac{7}{66} \end{pmatrix}, \mathbf{S^A} = \begin{pmatrix} -\frac{14}{209} \\ \frac{2}{39} \\ -\frac{1}{79} \\ -\frac{1}{98} \\ -\frac{5}{77} \\ -\frac{10}{139} \\ \frac{7}{73} \\ -\frac{1}{21} \end{pmatrix}, \mathbf{S^B} = \begin{pmatrix} -\frac{1}{71} \\ \frac{1}{1888} \\ -\frac{75}{173} \end{pmatrix}. \tag{21}$$

It can be verified that the state $\rho_{AB}(\alpha = 1/2)$ violates the 8-setting inequality with violation value as $\frac{313}{312} \backsimeq 1.0032$, where the 8 measurement settings of Alice can be characterized by Bloch vectors $\vec{x}_i$ $(i = 1, 2, ..., 8)$, which are

$$\mathbf{V} = \begin{pmatrix} \frac{5}{66} & -\frac{65}{82} & |z_1| \\ \frac{57}{65} & -\frac{38}{139} & -|z_2| \\ \frac{1}{41} & \frac{81}{82} & |z_3| \\ \frac{14}{17} & -\frac{112}{199} & |z_4| \\ \frac{18}{97} & -\frac{44}{51} & |z_5| \\ -\frac{45}{76} & -\frac{71}{134} & |z_6| \\ -\frac{32}{119} & \frac{2}{33} & -|z_7| \\ \frac{72}{85} & \frac{50}{113} & |z_8| \end{pmatrix}, \tag{22}$$

where the k-th row of the above matrix is understood to be $\vec{x}_k$, and $|z_k| = \sqrt{1 - v_{k1}^2 - v_{k2}^2}$.

Similarly, the 9-setting steering inequality is given by

$$\sum_{i=1}^{9}\sum_{j=1}^{3} s_{ij}\langle ab\rangle_{ij} + \sum_{i=1}^{9} s_i^A \langle a\rangle_i + \sum_{j=1}^{3} s_j^B \langle b\rangle_j \leq L, \tag{23}$$

with $L = 1$, and

$$\mathbf{S} = \begin{pmatrix} \frac{53}{521} & \frac{53}{902} & -\frac{47}{271} \\ -\frac{9}{280} & -\frac{13}{379} & -\frac{44}{273} \\ -\frac{39}{220} & -\frac{53}{419} & -\frac{17}{312} \\ \frac{34}{471} & -\frac{16}{339} & \frac{8}{55} \\ \frac{115}{2184} & -\frac{13}{426} & -\frac{63}{685} \\ \frac{23}{404} & -\frac{43}{354} & -\frac{34}{285} \\ \frac{29}{185} & -\frac{130}{873} & \frac{15}{289} \\ -\frac{26}{147} & \frac{2}{387} & -\frac{2}{111} \\ \frac{7}{132} & \frac{110}{353} & -\frac{21}{358} \end{pmatrix}, \mathbf{S^A} = \begin{pmatrix} -\frac{75}{974} \\ -\frac{23}{325} \\ -\frac{7}{305} \\ \frac{25}{389} \\ -\frac{11}{272} \\ -\frac{39}{751} \\ \frac{11}{467} \\ -\frac{1}{131} \\ -\frac{10}{359} \end{pmatrix}, \mathbf{S^B} = \begin{pmatrix} \frac{1}{564} \\ \frac{1}{161} \\ -\frac{26}{59} \end{pmatrix}. \tag{24}$$

One can show that the quantum violation for the state $\rho_{AB}(\alpha = 1/2)$ is $\frac{119}{116} \backsimeq 1.0258$, where the 9 measurement settings of Alice can be characterized by Bloch vectors $\vec{x}_i$ $(i = 1, 2, ..., 9)$, which are

$$\mathbf{V} = \begin{pmatrix} -\frac{272}{453} & -\frac{43}{124} & |z_1| \\ \frac{43}{165} & \frac{32}{115} & |z_2| \\ \frac{129}{161} & \frac{251}{439} & |z_3| \\ -\frac{7}{13} & \frac{25}{71} & -|z_4| \\ -\frac{68}{115} & \frac{37}{108} & |z_5| \\ -\frac{47}{131} & \frac{49}{64} & |z_6| \\ -\frac{123}{172} & \frac{36}{53} & -|z_7| \\ \frac{314}{315} & -\frac{3}{103} & |z_8| \\ -\frac{90}{541} & -\frac{89}{91} & |z_9| \end{pmatrix}. \tag{25}$$

Remark 4.— If the parameter α is not required to run over all the region of $[0, 1]$, one may also present other class of more efficient one-way steerable states. For the the simplest case, one may just select

$$f = 0, \tag{26}$$

i.e., the parameter is independent of α. Correspondingly from Eq. (15) one has the state as

$$\rho_{AB}(\alpha) = \frac{1}{4}\left(\mathbb{1} \otimes \mathbb{1} + \frac{1}{3}\,\sigma_3 \otimes \mathbb{1} - \frac{1}{2}\,\mathbb{1} \otimes \sigma_3 - \alpha \sum_{k=1}^{3} \sigma_k \otimes \sigma_k \right). \tag{27}$$

However, the matrix is a density matrix only if $\alpha \in [0, \frac{1}{18}(6+\sqrt{69})]$. For $\alpha = 1/2$, the state (27) is identical to the state (19), they all violate the 8-setting and the 9-setting steering inequality.

Eventually, we would like to mention that, due to the difficulty in numerical computations we are not able to obtain the optimal states for demonstrating the one-way EPR steering, which is a difficult problem. However, based on our result, the amount of quantum violation is much larger but achieved with fewer measurements in comparison to the previous result in [1]. To our knowledge, we do not know whether the quantum violation by inequality (23) could be observed with current quantum technology. However, we believe that this result would be interesting and helpful for the experimenters.

[1] Bowles, J., Vertesi, T., Quintino, M. T. & Brunner, N. One-way Einstein-Podolsky-Rosen Steering. *Phys. Rev. Lett.* **112**, 200402 (2014).
[2] Luo, S. Quantum discord for two-qubit systems. *Phys. Rev. A* **77**, 042303 (2008).
[3] Wiseman, H. M., Jones, S. J. & Doherty, A. C. Steering, Entanglement, Nonlocality, and the Einstein-Podolsky-Rosen Paradox. *Phys. Rev. Lett.* **98**, 140402 (2007).
